# Fe_3_O_4_ Magnetic Nanoparticles Obtained by the Novel Aerosol-Based Technique for Theranostic Applications

**DOI:** 10.3390/ma16196483

**Published:** 2023-09-29

**Authors:** Piotr Pawlik, Barbara Błasiak, Marcin Pruba, Arkadiusz Miaskowski, Oskar Moraczyński, Justyna Miszczyk, Boguslaw Tomanek, Joanna Depciuch

**Affiliations:** 1 Faculty of Production Engineering and Materials Technology, Częstochowa University of Technology, Armii Krajowej 19, PL-42-200 Częstochowa, Poland; m.pruba@gmail.com (M.P.); oskar.moraczynski@pcz.pl (O.M.); 2 Institute of Nuclear Physics, Polish Academy of Sciences, Radzikowskiego 152, PL-31-342 Krakow, Poland; justyna.miszczyk@ifj.edu.pl (J.M.); tomanek@ualberta.ca (B.T.); joanna.depciuch@ifj.edu.pl (J.D.); 3 Department of Applied Mathematics and Computer Science, University of Life Sciences in Lublin, Akademicka 13, PL-20-950 Lublin, Poland; arek.miaskowski@up.lublin.pl; 4 Department of Oncology, University of Alberta, 116 St & 85 Ave, Edmonton, AB T6G 2R3, Canada; 5 Department of Biochemistry and Molecular Biology, Medical University of Lublin, ul. W. Chodźki 1, PL-20-093 Lublin, Poland

**Keywords:** aerosol-based synthesis technique, TEM microstructure, Mössbauer spectroscopy, MTS assay, holotomography, MRI studies

## Abstract

This work is aimed at presenting a novel aerosol-based technique for the synthesis of magnetite nanoparticles (Fe${}_{3}$O${}_{4}$ NPs) and to assess the potential medical application of their dispersions after being coated with TEA-oleate. Refinement of the processing conditions led to the formation of monodispersed NPs with average sizes of ∼5–6 nm and narrow size distribution (*FWHM* of ∼3 nm). The NPs were coated with Triethanolammonium oleate (TEA-oleate) to stabilize them in water dispersion. This allowed obtaining the dispersion, which does not sediment for months, although TEM and DLS studies have shown the formation of small agglomerates of NPs. The different behaviors of cancer and normal cell lines in contact with NPs indicated the diverse mechanisms of their interactions with Fe${}_{3}$O${}_{4}$ NPs. Furthermore, the studies allowed assessment of the prospective theranostic application of magnetite NPs obtained using the aerosol-based technique, particularly magnetic hyperthermia and magnetic resonance imaging (MRI).

## 1. Introduction

Magnetic nanoparticles (NPs) have been utilized in many branches of modern industry. Their applications comprise manufacturing the pigments used in acrylic paints, producing electronic devices such as electrodes for batteries [1] and supercapacitors [2], hard drive elements [3], radio antennas, etc. They are also used in the acquisition of hydrogen, magnetic separation, the production of catalysts for chemical reactions [4], and as absorbents [5]. One of the most important challenges of our civilization is healthcare. Growing demands for personalized therapies and progress in diagnostics has resulted in the development of nanomedicine as a new field of science. Furthermore, therapeutic methods consider the use of magnetic nanoparticles as drug delivery carriers or agents in magnetic hyperthermia [6]. One of the magnetic materials fulfilling toxicological and biocompatibility requirements as well as being approved for medical applications is magnetite (Fe${}_{3}$O${}_{4}$) [7].

The antibacterial properties of iron-oxide NPs [8] and characteristics promoting the growth of skin cell cultures in vitro [9] are of great importance in modern medicine. They are frequently used as drug delivery carriers or in magnetic particle imaging (MPI). The functionalization of NPs by PEG and biotinylated Anti-EpCAM allows their use in the selective magnetophoresis of EpCAM-expressing pancreatic tumor cells [10]. The synthesis and in vivo imaging applications have been summarized in [11].

An important topic was discussed in [12], where the theoretical simulation of medical drug targeting was studied by modeling the flow of magnetite nanoparticles of various sizes in different magnetic fields. Based on the Circle of Willis model, the particle capture efficiency was assessed.

One of the diagnostic techniques is magnetic resonance imaging (MRI), where superparamagnetic NPs can be implemented as contrast agents. Application of the magnetic nanoparticles in MRI requires the fabrication of stable dispersions of NPs, which do not undergo agglomeration [13]. The aggregation may cause venous blockage hazard and noise in MRI signals, thus adversely affecting their use in biomedical applications. While MRI can be used for tumor diagnosis without contrast agents, their application enhances MRI diagnostic capability. Recently developed nanoparticles, in particular Fe${}_{3}$O${}_{4}$, have provided new opportunities and strategies in MRI applications and techniques (e.g., [14,15,16]), allowing MRI at the molecular level. The ideal MRI contrast agent requires a suitable size, narrow size distribution, excellent magnetic properties, low toxicity, and good biodegradability [17,18]. Unlike the standard contrast agents based on Gd${}^{3+}$, Iron-oxide meets most of these requirements—in particular, high biocompatibility and high *T*${}_{2}$ relaxivity. While standard contrast agents are based on passive accumulation in tumors due to the enhanced permeability and retention EPR effect, iron-oxide NPs can actively target cancer cells using specific ligands, thereby enhancing the specificity of diagnosis [19,20]. iron-oxide NPs can be easily functionalized with various ligands (e.g., antibodies or peptides), hence allowing targeted diagnosis of various types of cancer, including breast, stomach, colon, kidney, liver, prostate, or brain cancer. Furthermore, iron-oxide NPs have been used for other applications, such as the detection of brain inflammation or the early diagnosis of thrombosis [21,22,23,24]. Applications of theranostic agents, comprising both iron-based contrast and therapeutic agents, have shown improvements in early cancer detection and treatment [25]. Superparamagnetic NPs can also be utilized in labeling and tracking neutrophils using MRI [26].

Due to its magnetic properties, iron-oxides can also be used for hyperthermia treatment [27]. Cancer cells comprising iron-oxide NPs can be exposed to an alternating magnetic field delivering energy that converts into heat. It has been shown that the temperature can be increased to 45 °C for hyperthermia or to 60 °C and higher for thermoablation [28]. Heating impacts biochemical processes in the cells, causing alteration in their functions, including necrosis, toxicity, hypoxia, perfusion, and increased sensitivity to ionizing radiation [29]. Furthermore, hyperthermia was shown to enhance the cytotoxic effect of some chemotherapeutics [30,31]. Magnetic hyperthermia has already been used in clinics for the treatment of glioblastoma multiforme [32] and prostate cancer [33].

Consequently, magnetic nanoparticles are considered a very promising material in theranostic applications, and they contribute to increasing the patient’s comfort during diagnostics and treatment.

Numerous methods have been developed to produce magnetic NPs. An extensive overview of the synthesis techniques used for the fabrication of Fe${}_{3}$O${}_{4}$ nanoparticles and their application in photonics was presented in [34]. The chemical processes designed so far have shown a significant impact of manufacturing parameters on grain sizes and the size distribution of nanoparticles. These techniques can be divided into categories including solid, liquid, and gas synthesis routes. Among them, however, the liquid phase synthesis methods seem to be both the most diverse and efficient. These include techniques such as sol–gel co-precipitation, thermal decomposition, chemical reduction, microemulsion, and the use of microwaves [35,36,37].

An efficient and low-cost method used for the synthesis of Fe${}_{3}$O${}_{4}$ NPs is co-precipitation [38,39]. This involves dropwise addition of the basic solution into the solution of salts containing Fe${}^{2+}$ and Fe${}^{3+}$ ions. The particles are formed in the chemical reaction, for which the overall formula goes as follows:

(1) $${\mathrm{Fe}}^{2+}+{2\mathrm{Fe}}^{3+}+{8\mathrm{OH}}^{-}\to {\mathrm{Fe}}_{3}O_{4}+4H_{2}O$$

The precipitation of Fe${}_{3}$O${}_{4}$ occurs at a pH between 8 and 14 in a non-oxidizing environment. A solution containing salts with the stoichiometric ratio of (Fe${}^{3+}$/Fe${}^{2+}$) = 2:1 is used in this process. The magnetite is not very stable in the oxidizing aqueous solution, so the salt solution is bubbled with an inert gas (N${}_{2}$ or Ar). In adverse conditions (i.e., in the presence of oxygen), the magnetite transforms into maghemite (γ-Fe${}_{2}$O${}_{3}$). The best choice for clinical applications is the use of superparamagnetic iron-oxide NPs (magnetite or maghemite), which reveal a high magnetic moment. The main advantage of co-precipitation is the ability to produce a large quantity of NPs. However, since the growth of NPs is governed by the process kinetics, it is very difficult to control the particle size distribution [40,41]. According to LaMer concept [42], co-precipitation proceeds through two main stages: (i) abrupt nucleation of NPs when the species reach supersaturation and (ii) the slow growth of nuclei by diffusion of solutes to the crystal surface. To produce monodispersed iron-oxide NPs, it is required to separate these two stages during processing [43].

The variant of co-precipitation that partly allows fulfilling this requirement consists of the dropwise addition of the salts’ solution into the flask containing diluted hydroxide at the elevated temperature. Meanwhile, the hydroxide solution is bubbled with inert gas [44]. This technique allows obtaining a narrow size distribution of NPs with the average diameters of ∼12 nm. The main disadvantage of this precipitation procedure is the formation of a multiphase structure, where, except for precipitation of the main magnetite (Fe${}_{3}$O${}_{4}$) phase, a residue of maghemite (γ-Fe${}_{2}$O${}_{3}$), as well as NPs of non-stoichiometric magnetite, can be formed [45].

Several interesting approaches have been proposed to improve the physical conditions for the co-precipitation of nanoparticles [46]. A very efficient procedure for the synthesis of magnetic nanoparticles was presented in [47,48], where fast-flowing jets of component reagents were impinging inside the reactor chamber. The collision results in the formation of a thin liquid film in which the co-precipitation is initiated. This method allowed the production of cobalt ferrite (CoFe${}_{2}$O${}_{4}$) nanoparticles with an average diameter of ∼8 nm. Since this construction does not provide a gas-protective atmosphere, precipitation of the magnetite NPs is impossible using this technique.

A similar method was invented by Lin [49]. They used high-pressure swirl injectors for the precipitation of CaCO${}_{3}$ nanoparticles. This technique gave the possibility of size control. However, no data concerning obtained sizes were provided.

The advanced variant of co-precipitation was also presented in [50], which allows continuous production of magnetite nanoparticles based on the reaction between two reagents in a three-way mixer. Precipitated dispersion of magnetite NPs is subsequently mixed with citric acid fed to another mixer. The citric acid plays the role of neutralizer and causes the deagglomeration of NPs. It is possible to differentiate the sizes of resultant NPs using this technique.

The above-mentioned processes require either complex and expensive devices or cannot be used for producing magnetite nanoparticles. Therefore, we have developed a novel aerosol-based technique that overcomes these disadvantages (see Section 2.1). The idea was to create mists from low-concentration reagents using an inert low-pressure gas. The use of concentric nebulizers drawing the substrates from the containers by the inert gas is the novelty of this technique. Change in the gas pressure or concentration of reagents allows for control of the sizes of the obtained NPs. The contact between very small droplets of solutions results in the nucleation of small nanoparticles. The process involves the separation of the two stages of co-precipitation that were defined in the LaMer model [42], thus leading to more uniform NPs and their smaller sizes. This novel technique allows for maintaining the gas-protective atmosphere, producing small NPs, and can be easily transformed into the production line as it allows continuous synthesis of magnetite NPs. It is also a very cheap, efficient, and easy-to-use alternative to the more complex and less efficient synthesis techniques mentioned above, which is another advantage of this technique.

Our previous studies of co-precipitated magnetite nanoparticles subsequently coated with Triethanolammonium oleate (TEA-oleate) have shown their low cytotoxicity and the possibility to apply the NPs for MRI-guided hyperthermia [9]. In addition, the presence of nanoparticles in the cell cultures did not cause a significant increase in the number of apoptotic cells of the human dermal fibroblasts. It was shown that the presence of NPs has stimulated the proliferation of those cells, revealing their potential for application in regenerative medicine. Furthermore, the TEA-coated NPs formed stable uniform colloids (with the maximum studied concentration of about 0.1 g/mL), which did not sediment for several months. For this reason, we have applied the same procedure to stabilize nanoparticles. The current work aims to present the results of the synthesis and the properties of magnetite nanoparticles obtained by this novel aerosol-based technique.

## 2. Sample Preparation and Experimental Methods

The FeCl${}_{3}$·6H${}_{2}$O and FeSO${}_{4}$·7H${}_{2}$O salts, the 25% ammonia solution, and the citric acid (C${}_{6}$H${}_{8}$O${}_{7}$) used for the synthesis were purchased from Sigma-Aldrich® (Saint Louis, MO, USA) The oleic acid (C${}_{18}$H${}_{34}$O${}_{2}$) and triethanolamine ((CH${}_{2}$CH${}_{2}$OH)${}_{3}$N) used in the experiment were obtained from CHEMPUR® (Piekary Śląskie, Poland). The mixture of salts was diluted in 300 mL of deionized water. Separately, 18 mL of 25% NH${}_{4}$OH was mixed with 300 mL of H${}_{2}$O. The nanoparticles were synthesized at room temperature at two different processing conditions, using the novel aerosol-based technique that is discussed in detail in Section 2.1. The process parameters are collected in Table 1. This allowed obtaining two kinds of specimens: 1AeroNPs and 2AeroNPs.

### 2.1. Novel Aerosol-Based Technique for the Synthesis of Iron-Oxide NPs

**Figure 1:**
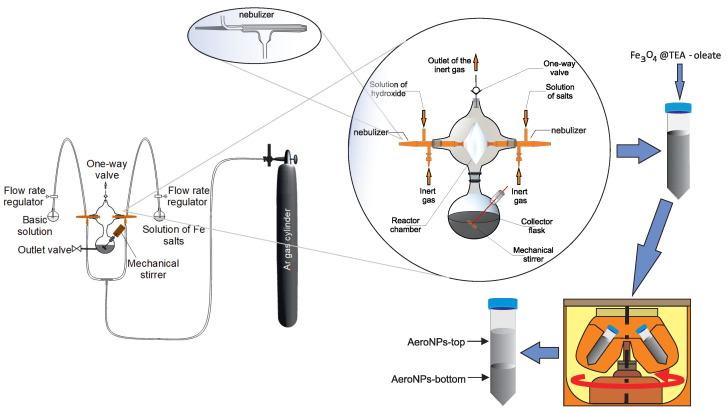
Schematic diagram of the aerosol-based process for precipitation of magnetite nanoparticles showing overall setup, the glassy reactor, TEA-oleate coating, and the obtaining of the “-top” and the “-bottom” fractions of NPs by centrifugation.

This novel procedure [51] can be considered an advanced variant of the co-precipitation technique (Figure 1). The principle of this synthesis lies in the formation of mists from salts and ammonia solutions by using glassy concentric nebulizers. The solutions are fed to them by lowering the gas pressures inside the nebulizers through the flow of an Ar gas, based on Bernoulli’s principle. As a result, the suction of the liquid solutions to the nebulizers takes place. Near their nozzles, the stream of argon atomizes the liquids, which are sprayed to the reactor chamber. The process is carried out in a four-neck glassy reactor kept at room temperature. The nozzles of the nebulizers face each other inside the glassy reactor. Such an arrangement allows the mixing of the mists inside the reactor chamber. Mixing salts and hydroxide mists, containing miniature droplets of both solutions, initiates the chemical reaction. The size of droplets and the concentration of reagents impact the speed of the reaction and the sizes of the formed nuclei. In the next stage, the dispersed nuclei of iron-oxide deposit on the inner surface of the reactor chamber. In their condensate, they undergo slow growth and are finally flushed into the collector flask. The water dispersion of NPs in the collector is mixed by a mechanical stirrer to reduce their excessive agglomeration at this stage of the process. The manufacturing of magnetite nanoparticles requires appropriate protection from oxygen during precipitation. The use of argon in the formation of mists additionally assures an appropriate atmosphere for the process.

For both kinds of specimen (1AeroNPs and 2AeroNPs), the solutions of salts and ammonia were fed to the reactor at constant dosage rates: 0.035 mL/s and 0.017 mL/s, respectively (Table 1). At the end of the process, the nanoparticles were separated by centrifugation (at 6000 rpm) and washed several times in deionized water to reach their neutral pH. Subsequently, the NPs were rinsed out with 6% of the citric acid and kept in an ultrasonic homogenizer for 1.5 min. After centrifugation, the NPs were further washed out twice in deionized water. Eventually, the NPs were dispersed in 50 mL of H${}_{2}$O.

### 2.2. Stabilization of NPs with Triethanolammonium Oleate (TEA-Oleate)

The TEA-oleate coating of the iron-oxide NPs is a process that was recently used to coat NPs fabricated by conventional co-precipitation [9]. Although the NPs obtained by the aerosol-based technique are quite stable in water, we have applied the same TEA-oleate coating to improve this effect. It consists of slow and alternating dropwise addition of 2 mL of oleic acid and 2.5 mL of triethanolamine to 50 mL of the water-dispersed magnetite NPs under vigorous mechanical stirring at 50 °C. The obtained mixture was left stirring at 50 °C for 2 h. The dispersion was subsequently subjected to centrifugation at 5000 rpm for 15 min, and then the supernatant was separated from the partly sedimented dispersion. This way, two kinds of specimens, designated as “-top” (supernatant) and “-bottom” (sediment), were obtained for 1AeroNPs and 2AeroNPs. The 1AeroNPs-top and 1AeroNPs-bottom were used only for the MRI studies. Except for MRI, in all the other studies the non-stabilized 1AeroNPs dispersion was used.

### 2.3. Materials Characterization

The microstructure and electron diffraction for the studied NPs were obtained using a transmission electron microscope TEM Jeol 2100 Plus (JEOL Ltd., Tokio, Japan). For TEM studies, the dispersions of NPs were diluted 500 times in deionized water and sonicated for 30 min. Subsequently, a droplet (of 5 μL size) of the diluted dispersion was applied to the carbon-coated copper grid (300 Mesh, Structure Probe Inc., West Chester, PA, USA), and left to dry. The application was repeated 5 times for each grid. The diameters of NPs were measured based on the TEM images using ImageJ software (National Institute of Mental Health, Bethesda, MA, USA). For this purpose, only clearly visible NPs (with well-defined grain boundaries) were selected. The diameters of approximately 200 nanoparticles were measured for each sample to prepare the histograms of the crystallite size distributions.

The X-ray diffraction patterns were collected using a Bruker D8 Advance X-ray diffractometer (Bruker AXS GmbH, Karlsruhe, Germany), working in Bragg–Brentano configuration with the use of Cu-K${}_{\alpha }$ radiation and an Ni filter of K${}_{\beta }$, located on the detector side (silicon strip LynxEye detector). The measurements were performed in the 2Θ range from 20 deg to 110 deg with steps of 0.02 deg and a time interval of 7 s per step. The Soller slit and anti-scatter slit were located correspondingly on the primary and secondary beams. Furthermore, the 0.6 mm slit was situated on the X-ray tube side. The Rietveld refinement of XRD patterns was performed using Diffrac.Topas 4.2 software. Prior to this analysis, the XRD pattern was measured for the LaB${}_{6}$ NIST 660a specimen to determine the emission profile of the X-ray tube and define the line widening related to the instrument characteristics. The Thompson–Cox–Hastings pseudo-Voigt function was used to define the type of the line profile, based on the emission profile and shape of the diffraction line determined using the LaB${}_{6}$ sample. These initial parameters were used in the Rietveld analyses performed on the XRD spectra measured for the studied NPs.

The magnetic properties of the specimens were measured using a LakeShore 7307 VSM magnetometer (Lake Shore Cryotronics, Inc., Westerville, OH, USA), operating at room temperature in magnetic fields up to 2 T. The studied dispersions were dried in a vacuum oven at 60 °C for 24 h. As the 1AeroNPs sample was initially a water dispersion of nanoparticles (without further coating with TEA-oleate), consequently the powder specimen was obtained. On the other hand, drying of the 2AeroNPs-top and 2AeroNPs-bottom specimens resulted only in the removal of the water, while TEA-oleate remained on the surface of the NPs. Then each specimen was immobilized in the VSM holder for magnetic measurements.

The phase structure and magnetic ordering of NPs were confirmed by Mössbauer spectra analysis. The spectra were recorded at room temperature by the use of the Integrated Mössbauer Spectroscopy Measurement System (Elektronika Jadrowa, Kraków, Poland), operating in transmission mode. The ${}^{57}$Co:Rh γ-rays source with an activity of ≈50 mCi was used for measurements. The spectra were collected using a multichannel analyzer with a gas proportional counter used as a detector. For this purpose, 14.4-keV γ-ray pulses were selected. The velocities of the Mössbauer source were calibrated with the use of 10-μm-thick α-Fe foil at room temperature. Numerical analysis of the Mössbauer spectra was performed by the use of the WinNormos-for-Igor Software Package (Wissenschaftliche Elektronik GmbH, Starnberg, Germany).

Dynamic light scattering (DLS) studies were performed on dispersions of the NPs using a Zetasizer Lab instrument (Malvern Panalytical Ltd., Malvern, UK). The samples for DLS measurements were prepared by making a 1:1000 dilution of the initial dispersions in deionized water. Before the measurements, the DLS specimens were placed in the ultrasonic bath for 15 min.

The heating efficiency of NP dispersions in alternating magnetic fields was studied using a magneTherm system (nanoTherics, Staffordshire, UK). The calorimetric experiments were conducted under non-adiabatic conditions on 2-mL water dispersions of the 2AreoNPs-top and 2AeroNPs-bottom samples with concentrations of *d${}_{t}$* = 197.94 mg/mL and *d${}_{b}$* = 231.70 mg/mL, respectively. The samples were exposed to the magnetic fields, *H* = 5 kA/m and *H* = 15 kA/m, of various frequencies (*f*), namely *f* = 109, 177, 266, and 532 kHz. The time dependencies of the temperature, *T*(*t*), were recorded with a time gap of 0.5 s.

### 2.4. Characterization of Interactions between NPs and Biological Material

#### 2.4.1. Cell Lines and MTS Assay

To determine a nontoxic concentration of NPs for selected cell lines, the water dispersions of NPs, particularly 20 μg/mL, 50 μg/mL, 100 μg/mL, 150 μg/mL, and 200 μg/mL, were prepared. The commercially available cell lines U118 glioblastoma cancer (ATCC ®(Manassas, VA, USA), No. HTB-15TM) and CHO-K1 cells of the ovary of a Chinese Hamster (ATCC®-CCL-61) (representing normal cells) were used for estimation of the viability of the cells. CHO-K1 cells are considered the most popular and sensitive cell lines for cytotoxicity screening. U-118 cells were maintained in Dulbecco’s modified Eagle’s medium (DMEM, ATCC®, No. 30-2002), while the CHO-K1 cells were kept in F-12K medium (ATCC®, No. 30-2004), respectively. The cells were placed in a 25 cm${}^{2}$ cell culture flask (TPP®, nr 90026) and kept in a humidified incubator at 37 °C filled with the atmospheric gas containing 5% CO${}_{2}$. The culture was topped up with 10% fetal bovine serum (FBS, Gibco®, nr 10270106) (Thermo Fisher Scientific, Waltham, MA, USA). When a 70% confluent monolayer of cells was reached, it was fully detached with the use of trypsin solution EDTA 1X (Millipore Sigma nr 59430C) (Merck, Darmstadt, Germany). The trypsin-containing suspension of cells was then deactivated by its dilution in fresh culture medium and centrifuged (for 3 min at 700 rpm) in 15 mL conical test tubes. Then, the cell sediment was again suspended in the fresh culture medium to assess the cell number. The process was performed 5 times. Subsequently, the cells were seeded within 96-well plates. After 24 h, the 10-μL dispersion of nanoparticles was added to those cells. The MTS tests were carried out after 3 h and 24 h after the addition of NPs. They were performed 3 times for each concentration and each type of NPs. The results were averaged and presented together with a standard deviation. Furthermore, the results were compared with the control specimen to reveal significant differences between these specimens.

#### 2.4.2. Holotomographic Microscopy

For the holotomographic studies, the cells were seeded within another 96-well plate made of SBS material that allows real-time visualization of cell behavior in the presence of NPs. For those cells, 80 μL of the fresh culture medium was added. The studies were carried out using a 3D Cell Explorer-Fluo (Nanolive, Tolochenaz, Switzerland) holotomographic microscope, enabling ×60 magnification. In this study, a green-light laser with a wavelength of λ = 520 nm, irradiance of 0.2 mW/mm${}^{2}$, and a field depth of 30 μm was used. These conditions were set to determine the refracting indexes (RI) required for the reconstruction of the 3D holotomographic images. The holograms are acquired through the interaction between the light passing through the specimen and the reference beam. The rendering of holograms was carried out using STEVE® software for the reconstruction of 3D images.

### 2.5. Measurements of Relaxivity in MR Experiment

The spin–lattice *T*${}_{1}$ and spin–spin *T*${}_{2}$ relaxation times of the aqueous dispersions of NPs were measured using a 9.4 T/21 cm bore magnet (Magnex Scientific LTD, Oxford, UK) and a Bruker console (Bruker BioSpin, Ettlingen, Germany). A transmit/receive radio frequency volume birdcage coil was applied to excite protons and obtain an MR signal. For the *T*${}_{2}$ measurements, a single-slice multi-echo pulse sequence was used with the following pulse parameters: repetition time (*TR*)—5 s, matrix size—128 × 128, field of view (*FOV*)—3 cm × 3 cm, slice thickness—2 mm, echo train length (*ETL*)—128, and echo spacing (*ESP*)—4 ms. The *T*${}_{2}$ relaxation times were calculated using a single exponential fitting of the echo train using Marevisi software (Institute for Biodiagnostics, National Research Council, Canada). For the *T*${}_{1}$ measurements, the *TRUE FISP* method was used with the following pulse sequence parameters: *TR*—3 s, *TE*—1.5 ms, matrix size—128 × 128, FOV—3 cm × 3 cm, slice thickness—3 mm, 60 frames × 4 segments, and segment time—192 ms. The *T*${}_{1}$ relaxation times were calculated using a single exponential fitting of the data (MATLAB 9.4 “1sqcurvefit”) for different concentrations of NPs [52].

## 3. Results and Discussion

### 3.1. Tem Studies

**Figure 2:**
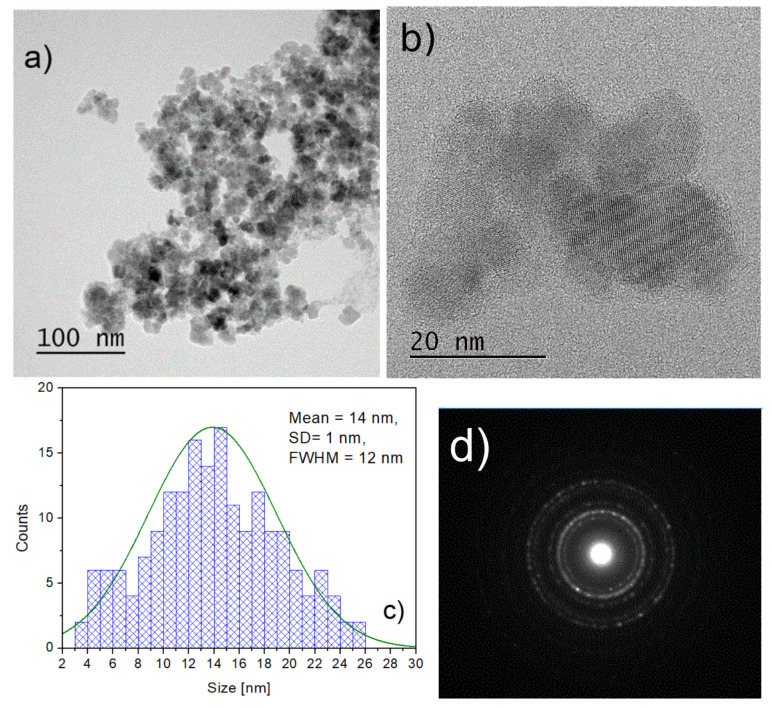
TEM micrographs of the 1AeroNPs nanoparticles obtained using the aerosol-based technique (**a**,**b**), histogram of the crystallite size distribution (**c**), and the electron diffraction pattern for the studied NPs (**d**).

In Figure 2a,b the microstructure of the 1AeroNPs specimen is presented, together with a histogram of the crystallite size distribution constructed from measurements made on TEM images (Figure 2c). Here, a relatively wide size distribution of NPs was observed. The maximum in the size distribution corresponds to nanoparticles with a diameter of ∼14 nm. Although a large number of NPs had diameters larger and smaller than 14 nm, this distribution is better than that for the conventionally co-precipitated magnetite NPs. However, further refinement of this process was necessary to obtain a narrower distribution of particle sizes (one should notice that the average crystallite diameters of NPs obtained using the standard co-precipitation method varied from 10 to 20 nm [9,53], and NPs have a relatively wide distribution of sizes). The electron diffraction studies confirmed the diversified microstructure of the specimen (Figure 2d), where some intensive diffraction spots attributed to the presence of larger NPs are visible on top of the diffraction rings coming from the smaller nanoparticles.

**Figure 3:**
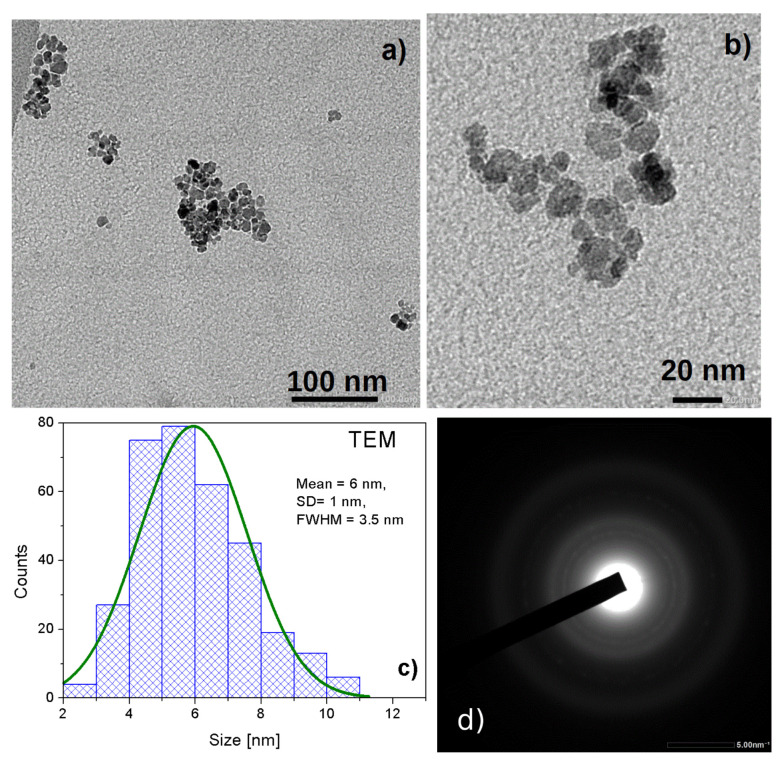
TEM micrographs of the 2AeroNPs-top nanoparticles obtained using the aerosol-based technique (**a**,**b**), histogram of the crystallite size distribution (**c**), and the electron diffraction pattern for the studied NPs (**d**).

**Figure 4:**
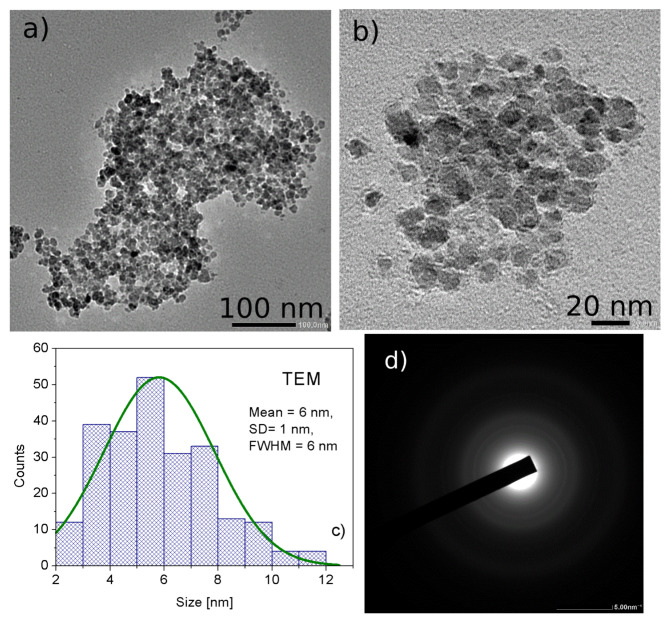
TEM micrographs of the 2AeroNPs-bottom nanoparticles obtained using the aerosol-based technique (**a**,**b**), histogram of the crystallite size distribution (**c**), and the electron diffraction pattern for the studied NPs (**d**).

Appropriate adjustment of the processing conditions, as shown in Section 2 for the 2AeroNPs specimen, allowed the fabrication of ultra-small superparamagnetic nanoparticles (USPION), as presented in Figure 3. This was possible by changing the synthesis parameters, further TEA-coating, and the centrifuge separation of the dispersion into two fractions. In the case of the 2AeroNPs-top specimen, the maximum of the distribution is located at ∼5–6 nm. The NPs in this fraction were not larger than 11 nm and were far smaller than those obtained using the standard co-precipitation technique. The full width at half maximum (*FWHM*) of its distribution reached 3.5 nm, proving the effectiveness of this process. The TEM images taken for both 2AeroNPs-top and 2AeroNPs-bottom specimens (Figure 3a,b and Figure 4a,b) have shown the formation of agglomerates of magnetite NPs. However, their sizes are different depending on the fraction of the nanoparticles. In particular, the TEM images of the 2AeroNPs-bottom have shown the presence of larger agglomerates (up to ∼300 nm) constituting this specimen (Figure 4a). This might be caused by the fact that this particular dispersion is obtained as a partly sedimented fraction after centrifugation. In addition, the particle size distribution is wider and its *FWHM* reaches 6 nm (Figure 4c), though the maximum of the distribution corresponds to ∼5–6 nm (as in the case of the 2AeroNPs-top specimen). The electron diffractions for both specimens (the 2AeroNPs-top, Figure 3, and 2AeroNP-bottom, Figure 4d) form uniform rings characteristic of the homogeneous and very small nanoparticles.

### 3.2. X-ray Diffraction

**Figure 5:**
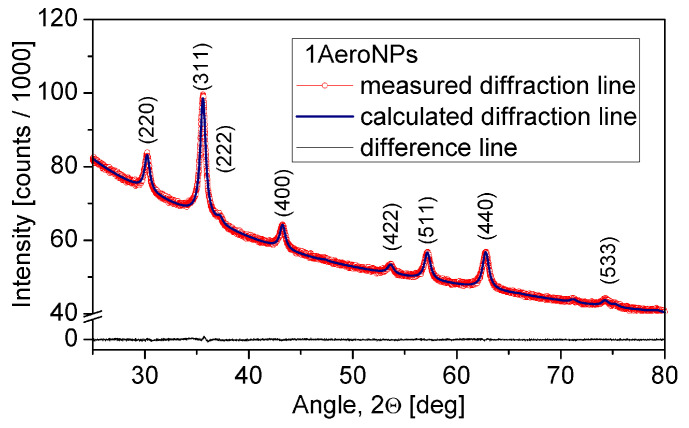
XRD scan and its Rietveld refinement for the 1AeroNPs specimen.

**Figure 6:**
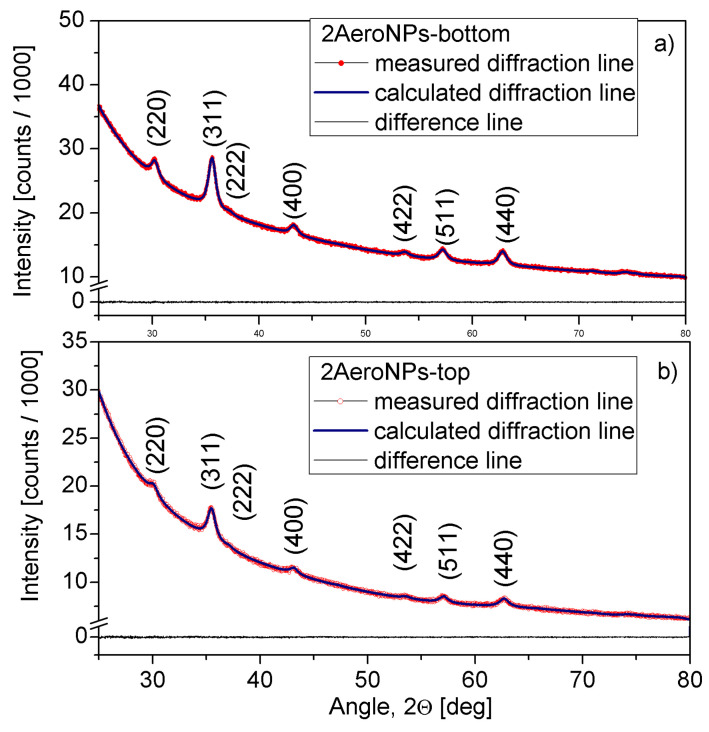
XRD scans for the 2AeroNPs-bottom (**a**) and 2AeroNPs-top (**b**) specimens, together with the Rietveld refinement lines.

The XRD scans measured for all specimens were subjected to Rietveld refinement. Studies have shown the presence of diffraction peaks characteristic of the Fe${}_{3}$O${}_{4}$ phase (Figure 5 and Figure 6). The *R${}_{exp}$*, *R${}_{wp}$*, and *GOF* values collected in Table 2 are the Rietveld refinement parameters that prove a good fit for the experimental scans. In all investigated specimens, the slightly smaller unit cell parameter *a*, compared with that for bulk magnetite (*a* = 8.397 Å—also used as a starting value in the Rietveld refinement) can be attributed to the nanocrystalline microstructure of the samples. However, one can consider that it might only be a result of the phase transformation from the magnetite to the maghemite phase (having smaller unit cell parameter *a*), occurring on the surface of NPs.

The calculated *L${}_{vol}$* for all studied specimens are also collected in Table 2. *L*${}_{vol}$ is a factor that describes the volume-weighted coherently diffracting domain sizes and can be considered as an indicator of the average crystallite diameters. In all cases, the *L*${}_{vol}$ are close to the maxima of the particle size distributions obtained from the TEM studies.

### 3.3. The Effect of Magnetic Field on the Dispersion of NPs Stabilized by the TEA-Oleate/DLS Studies

**Figure 7:**
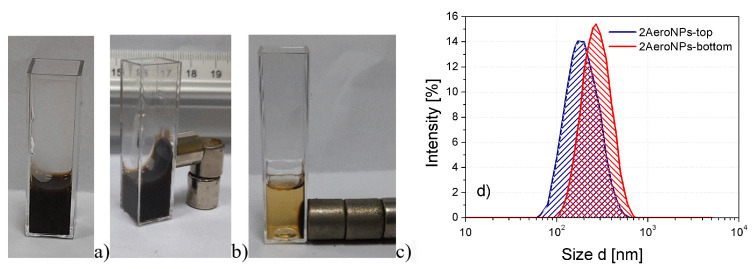
The 2AeroNPs-top dispersion of magnetite nanoparticles stabilized by TEA-oleate (**a**), the sample attracted by a magnet for 1 min (**b**) and 24 h (**c**), and the DLS hydrodynamic sizes for 2AeroNPs-top and 2AeroNPs-bottom specimens (**d**).

NPs coated with TEA-oleate are hydrophilic and form uniform dispersion in water, which is stable and does not sediment for months. Their behavior in the presence of an external magnetic field is shown in Figure 7 for the 2AeroNPs-top specimen. Applying the magnetic field resulted in the separation of NPs after approximately 1 day (Figure 7c). The dispersion returns to its previous state after the removal of the magnet and a firm shake. The DLS studies carried out on TEA-oleate-coated 2AeroNPs-top suggest a formation of small agglomerates of superparamagnetic NPs (with an average hydrodynamic size of ∼160 nm) (Figure 7d), which is consistent with results obtained by TEM studies (Figure 3a). In addition, in the case of the 2AeroNPs-bottom, the peak at dynamic light scattering particle size distribution reaches 340 nm, which is similar to the sizes of agglomerates observed using TEM (Figure 4a). Although the use of TEA-oleate resulted in good resistance against sedimentation, further studies are needed to confirm the aggregation stability by performing measurements after some time.

### 3.4. Mössbauer Spectra Analysis

In the current studies, all specimens were placed in a vacuum dryer, and the water was evaporated. However, it was impossible to obtain fully dry nanoparticles as the 2AeroNPs-top and 2AeroNPs-bottom samples were left coated with liquid TEA-oleate. This affected the shapes of the Mössbauer spectra.

**Figure 8:**
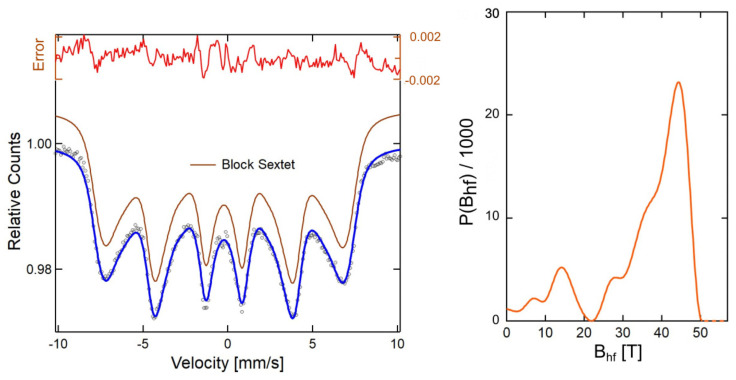
Room temperature Mössbauer spectrum measured for the 1AeroNPs sample (black circles), together with the hyperfine field distribution (light red line) corresponding to the fitting block sextet line (brown). The error line (dark red) represents a difference between the fitting line and the measurement points. Blue line-the complete fit.

**Figure 9:**
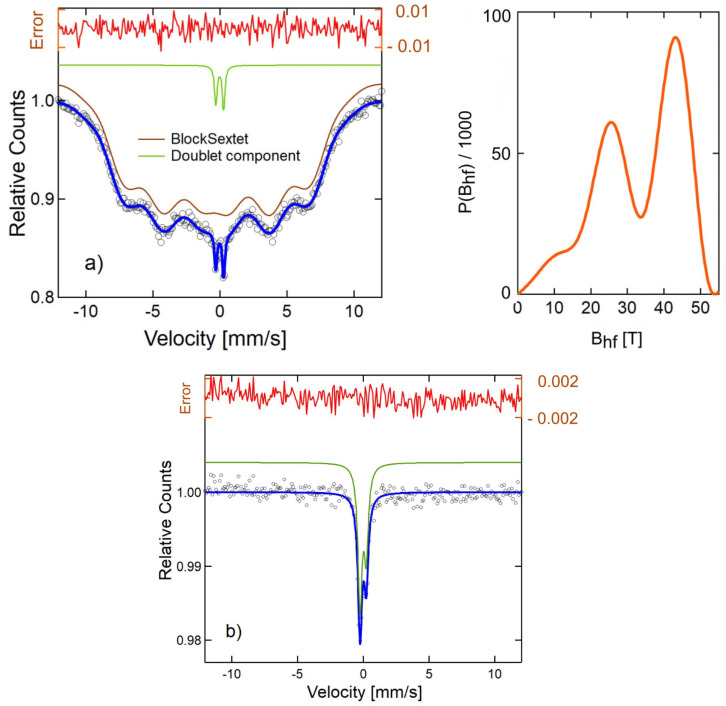
Room temperature Mössbauer spectra, measured for the 2AeroNPs-bottom sample (black circles) together with the fitted constituent lines (green and brown) and hyperfine field distribution (light red line) (**a**) and for the 2Aero-top sample (**b**) fitted with a single doublet line (green line). The error lines (dark red) represent the differences between the fitting line and the measurement points.

In the case of 1AeroNPs (Figure 8), a wide Mössbauer sextet line was measured. In this case, it was possible to fit the experimental spectrum using a block sextet line corresponding to the hyperfine field distribution, as also presented in Figure 8. The shape of the Mössbauer spectrum suggests the existence of some interactions between the superparamagnetic NPs forming large agglomerates and thus revealing ferrimagnetic behavior. This effect is also partly visible in the case of the 2AeroNPs-bottom sample. Here, except for a very wide sextet line, an additional doublet component corresponding to the presence of separated superparamagnetic NPs was observed (Figure 9a). As was shown in [3], collective interaction between magnetic nanoparticles in their agglomerates causes an increase in the fluctuation time of the magnetic moment. This effect has its feedback in the shapes of the Mössbauer spectra. The superparamagnetic state of NPs is more pronounced in the case of the 2AeroNP-top sample (Figure 9b), where its Mössbauer spectrum was well fitted using a single doublet line.

### 3.5. Room Temperature Magnetic Measurements

As was shown in [54], an MPMS-3 (Quantum Design) equipped with a quantum interference device (SQuID) is suitable for studying the behavior of nanoparticles dispersed in a liquid medium. Here, the superconducting magnet generates a uniform magnetic field on a larger scale. Furthermore, the sample does not move in the magnetic field. It allows for assessing the impact of size and interactions between NPs dispersed in a liquid medium based on the shapes of M(H) curves.

**Figure 10:**
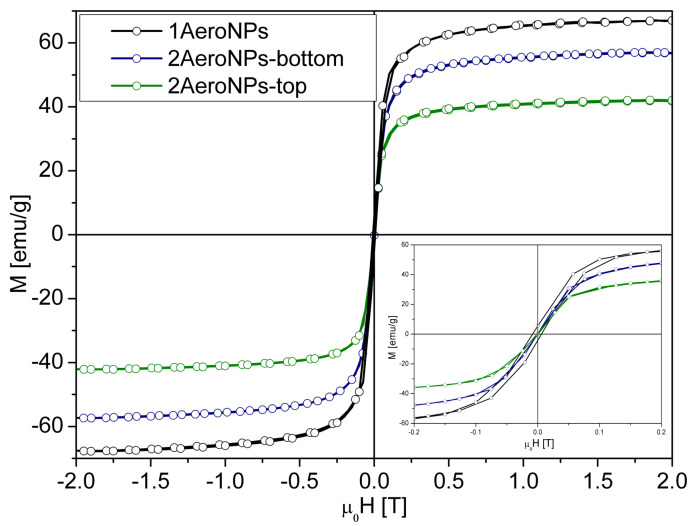
Room temperature hysteresis loops measured for all investigated specimens.

On the other hand, the VSM magnetometry used in our measurements does not allow such studies due to its characteristics (the sample is vibrating, and the horizontal magnetic field is uniform in a small area). However, our objective was not to define the properties of separated NPs or to study their interactions in a liquid medium. Instead, our goal was to obtain information about the agglomerates themselves (while coated in TEA-oleate or non-coated), as their magnetic characteristics will impact their performance in MRI and hyperthermia studies. The soft ferrimagnetic properties of the studied specimens were revealed by the measurements of the hysteresis loops at room temperature (Figure 10). The ferrimagnetic state might be caused by the drying of the specimens before measurement, thus causing agglomeration and subsequent magnetic interactions between superparamagnetic NPs. The highest saturation magnetization (*M${}_{s}$* = 66.8 emu/g) was measured for the 1AeroNPs sample, which is smaller than that for the bulk magnetite at room temperature (92 emu/g) [55,56,57]. For the 2AeroNPs-bottom and 2AeroNPs-top samples, the *M${}_{s}$* was even smaller. This might be related to the presence of TEA-coating on top of NPs and their agglomerates after drying, which causes a reduction of the interactions between NPs. One can expect that the existence of larger agglomerates in the 2AeroNPs-bottom sample has also contributed to the higher saturation magnetization than that measured for the 2AeroNPs-top. Furthermore, changes in densities of the TEA-oleate-coated specimens in comparison to the non-coated dry 1AeroNPs sample can also impact the value of *M${}_{s}$* (coating reduces the density of the samples).

### 3.6. Mts Assay

**Figure 11:**
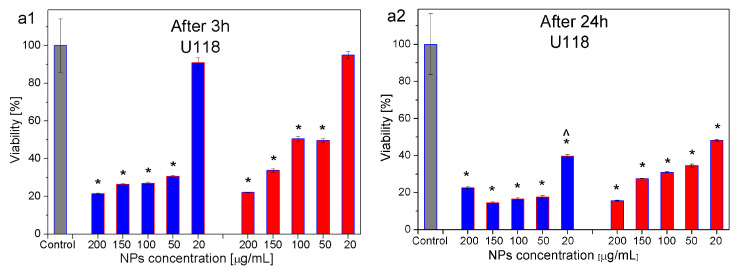

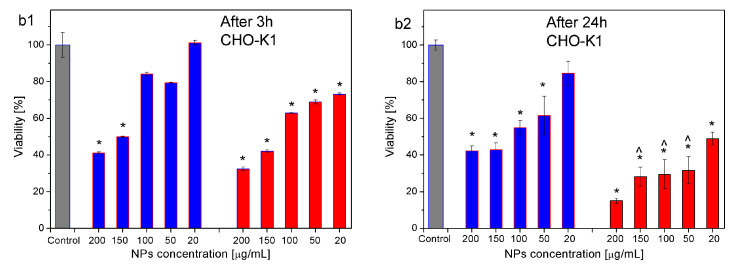
Viability of U118 glioblastoma cancer cells (**a1**,**a2**) and CHO-K1 normal cells (**b1**,**b2**) cultured for 3 h (**a1**,**b1**) and 24 h (**a2**,**b2**) with the addition of 2AeroNPs-bottom (blue bars) and 2AeroNPs-top (red bars) nanoparticles of different concentrations. Here, (*) p< 0.05 vs. control sample (black bar), (∧) p< 0.05 vs. the same sample but after 24 h.

The changes in the viability of U118 glioblastoma cancer cells (Figure 11a) and CHO-K1 normal cells (Figure 11b), after the addition of 2Aero-top and 2Aero-bottom nanoparticles, were investigated with the use of MTS assay. As mentioned previously, the MTS assay was performed for 5 different concentrations of NPs of 200 μg/mL, 150 μg/mL, 100 μg/mL, 50 μg/mL, and 20 μg/mL. To reveal the short- and long-term impact of NPs on the cells, the viability tests were performed after 3 h and 24 h. To determine statistical significance in the differences of the cell viability, one-way ANOVA with Tukey’s post hoc test was performed with the use of Past 4.0. software. In our studies, the statistical significance was assessed by calculating the *p*-values, which were lower than 0.05. The results were compared with the control sample as well as with the same sample after 24 h of incubation.

The MTS assay showed no significant changes in the viability of the U118 cells after their contact for 3 h with magnetite nanoparticles (stabilized by TEA-oleate) of the concentration of 20 μg/mL. Similar results were obtained for both the 2AeroNPs-top and the 2AeroNPs-bottom (Figure 11(a1)) (after 3 h of exposure). The MTS test showed that the viability of the U118 cells decreases with the increase of the concentration of NPs in the dispersion. For 2AeroNPs-bottom (blue bars in Figure 11(a1)), the cell viability varied from 31% to 22% when the concentration of NPs rises from 50 μg/mL to 200 μg/mL. On the other hand, for the 2AeroNPs-top nanoparticles (red bars in Figure 11(a1)), the mortality of cells was smaller and varied from 50% to 78% for the same changes in concentration of NPs (from 50 μg/mL to 200 μg/mL). Significant differences were visible when comparing the tests performed on U118 cells after their contact with NPs for 3 h and 24 h. For the cells tested after 24 h (Figure 11(a2)), significant changes in the viability of the cells were visible, even for an NP concentration of 20 μg/mL added to this culture. In particular, in the case of 2AeroNPs-bottom nanoparticles, when the concentration of NPs increased from 20 to 50 μg/mL a significant decrease in the viability of U118 cells was observed. However, with the increase of NP concentration from 50 to 150 μg/mL, only small changes in their viabilities were noticed. For dispersions containing 2AeroNPs-top (red bars in Figure 11(a2)), a gradual decrease in the viability of cells from 36% to 17% was detected, respectively, for concentrations of NPs from 50 μg/mL to 200 μg/mL. After 24 h, the largest changes in the viability of cells (approximately 20–25% compared to those in contact with NPs for 3 h) occurred for the 2AeroNPs-top specimen and were dependent on the concentration of NPs added to the cells culture.

In the case of CHO-K1 normal cells cultured for 3 h with the 2AeroNPs-bottom nanoparticles, a significant decrease of cell viability was detected for two of the highest concentrations of nanoparticles: 200 μg/mL and 150 μg/mL (blue bars in Figure 11(b1)). On the other hand, in the case of 2AeroNPs-top specimens, an increase in cell mortality was noticed for all tested NP concentrations (red bars in Figure 11(b1)). After 24 h, the most visible changes in the cell viability (approximately 20–25% compared to those cells cultured for 3 h with the addition of NPs) were present for the 2AeroNPs-top sample (red bars in Figure 11(b2)). In the case of 2AeroNPs-bottom, very small changes in the cells’ mortality were detected after 3 h and 24 h for the concentration of NPs of 20 μg/mL. Also for other concentrations of NPs, when comparing the results obtained after 3 h and 24 h of culture, the differences in the viabilities of the cells were not very significant (Figure 11(b2)).

An important outcome of the current studies is a higher mortality of the cancer cells after the addition of NPs to their culture, while only a little damage to the healthy cells was observed. This suggests that studied magnetite nanoparticles could be used in anticancer therapies, although further in-depth investigations have to be continued.

### 3.7. Holotomographic Studies

**Figure 12:**
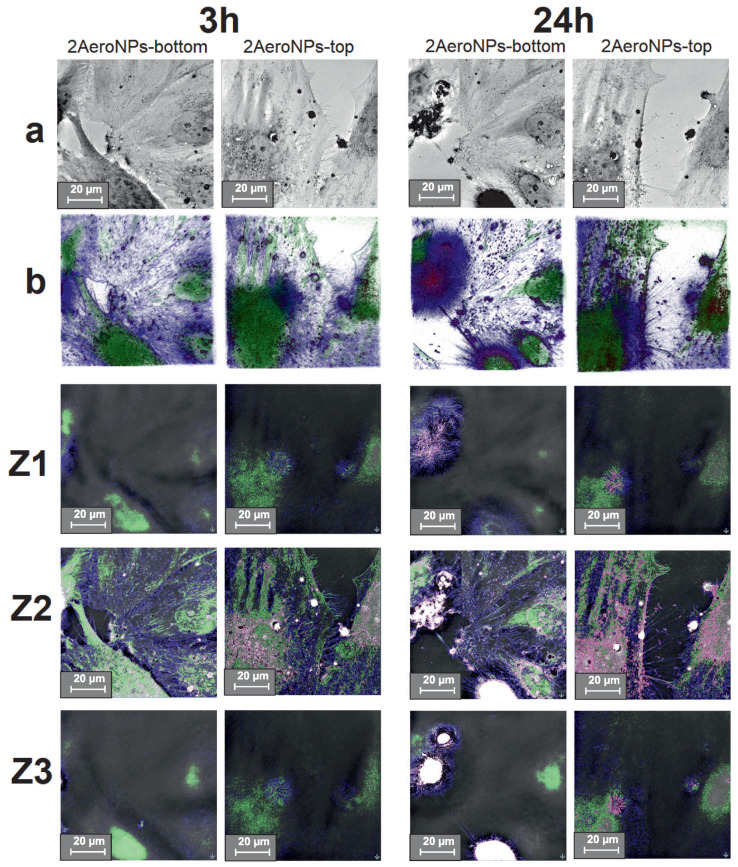
Holotomographic images of U118 cells cultured with the 2AeroNPs-top and 2AeroNPs-bottom dispersions with a concentration of 20 μg/mL (a); 3D images reconstructed from RI differences between NPs (red), nucleus and cytoplasm (green), and cell membrane (blue) (b); Z1, Z2, and Z3: holotomographic images of the respective layers. Images were taken after 3 h and 24 h with a scale of 20 μm.

**Figure 13:**
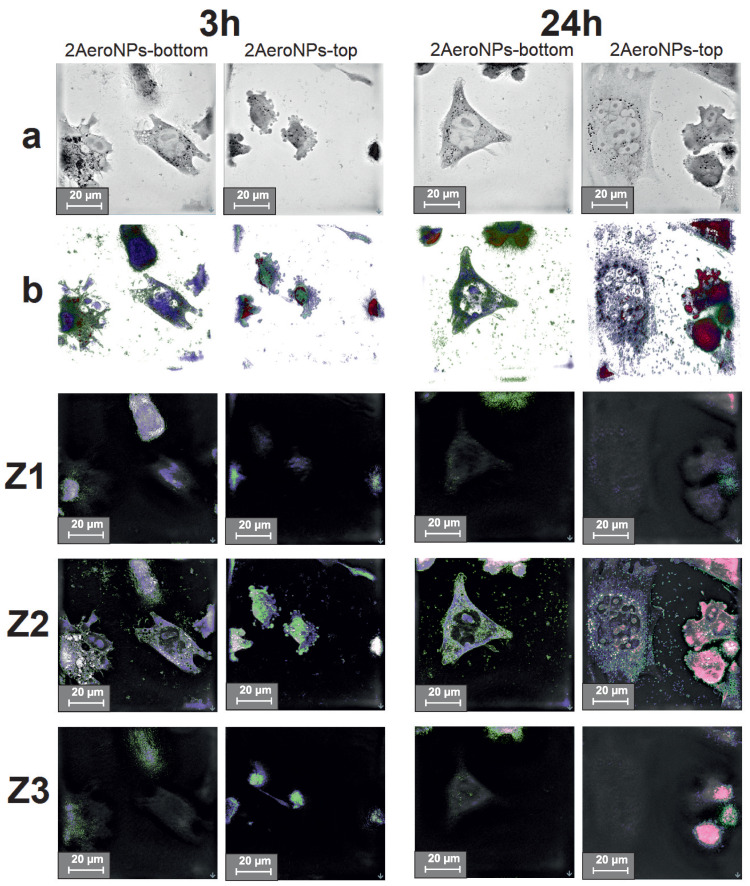
Holotomographic images of CHO-K1 cells cultured with the 2AeroNPs-top and 2AeroNPs-bottom dispersions with a concentration of 20 μg/mL (a); 3D images reconstructed from RI differences between NPs (red), nucleus and cytoplasm (green), and cell membrane (blue) (b); Z1, Z2, and Z3: holotomography images of the respective layers. Images were taken after 3 h and 24 h with a scale of 20 μm.

To determine the effect of NPs on the morphology of cells, holotomographic images were taken for U118 and CHO-K1 cells cultured with the smallest concentration of NPs (precisely for 20 μg/mL). Moreover, to show whether the NPs were accumulated outside or inside the cells, the holotomographic cross-sections were made at approximately 0 μm (Z1), 7 μm (Z2), and 15 μm (Z3) for each analyzed sample. Those designated depths correspond to the top of the cells (visualizing the outside membrane of the cells—Z1), the center of the cells (visualizing the inside area of the cells—Z2), and the bottom of the cells—Z3. Additionally, the nanoparticles, the cell membrane, and the nucleus with cytoplasm were marked with different colors in the homolographic images Z1–Z3, based on the differences in their refraction indexes (RI) (Figure 12 and Figure 13), to show places of accumulation of NPs.

The obtained holotomographic images show that the U118 cells were alive, even after 24 h from the moment of NP addition to their culture. This applies to both types of tested NPs. In the case of 2AeroNPs-bottom dispersion, the formation of large agglomerates of nanoparticles was seen after 24 h. Furthermore, after 24 h the cells cultured with both 2AeroNPs-bottom and 2AeroNPs-top formed stress fibers, which may come from the accumulation of a large number of NPs (Figure 12a). Holotomographic images obtained after the reconstruction (concerning the differences in RI for nanoparticles and cell structures) clearly show that the volume of nanoparticles accumulated inside U118 cells increased with time (Figure 12b). In addition, for both studied types of nanoparticles (after 24 h), it was clear that their preferred place of accumulation was the membrane surrounding the cell nucleus. The cross-section images (Z1–Z3) reveal that the nanoparticles penetrated the interior of the cells, even after 3 h of their presence in the U118 culture. This trend was visible for both studied dispersions. However, after 24 h it was shown that the 2AeroNPs-bottom and 2AeroNPs-top nanoparticles were located both on the upper and lower parts of the cell membrane. In addition, the 2AeroNPs-top nanoparticles form aggregates that are visible after both 3 h and 24 h of culture (Figure 12b,Z1–Z3).

Considering the interaction of NPs with normal cells, no significant changes in their morphology have been observed for CHO-K1 cells cultured with the addition of 2AeroNPs-bottom nanoparticles. Although, in the case of the 2AeroNPs-top sample, the nanoparticles caused membrane budding after only 3 h of their culture; however, images taken after 24 h revealed a lack of pathological changes (Figure 13a). The reconstruction of holotomographic images clearly shows that the accumulation of nanoparticles in CHO-K1 cells increased over time. Moreover, it was seen that after 3 h, the nanoparticles did not take any preferred accumulation sites, while after 24 h the 2AeroNPs-top nanoparticles were accumulated mainly around the cell nucleus (Figure 13b). Depth images (Figure 13Z1–Z3) revealed, that after 3 h, both types of nanoparticles were located inside the cells, and for the 2AeroNPs-bottom, the NPs additionally were accumulated outside the cell on the upper layer of the cell membrane. Eventually, after 24 h, both types of nanoparticles were located inside the cells, but also outside, on their lower parts.

One can assume that particles show a certain level of toxicity in all concentrations above 20 μg/mL for both U118 and CHO-K1 cells, even after 3 h of incubation (Figure 11). It is also presented in Figure 12 and Figure 13, for cells incubated for 3 h with the addition of NPs, that the nanoparticles were agglomerating, even for the concentration of 20 μg/mL. This suggests that the toxic effect of the NPs could also be caused by the formation of these agglomerates. However, more extensive studies of the toxic effect of NPs on normal and cancer cells as well as the mechanism of interactions between cells and NPs have to be carried out and will be the next step of our research. It is well known that the most important factors that affect the internalizations of nanoparticles with the cell cultures are shape, size, and the surface charge of NPs, as well as the type of cells used [58,59]. Small positively charged NPs have shown increasing dynamics of internalizations in comparison with negatively charged NPs of larger diameters. This is explained by the fact that the cell membrane has a negative charge, thus attracting positively charged NPs [60]. In our study, we used 2AeroNPs-top and 2AeroNPs-bottom samples where the sizes and shapes of NPs are very similar. Therefore, we consider the type of cells as the most important factor. Indeed, smaller internalizations of the NPs with normal cells were shown. This could be explained by differences in the metabolism between cancer and normal cells [61]. Carelo et al. showed in the TEM images of MCF-7 cells that macropinocytosis uptake and clathrin-mediated internalization depended on the nanoparticle’s aggregate sizes. However, no changes in the morphology, cell cycle, or organization of the cytoskeleton have been noticed [62]. On the other hand, Zhang et al. showed that iron-oxide nanoparticles were internalized by the cancer and non-cancer cells with the same vesicular transport mechanism [63]. Our holotomographic studies have also shown that investigated NPs were visible inside both normal and cancer cells. However, in the case of normal cells, the toxic effect is lower. Taking into account these results, we believe that the nanoparticles synthesized using the aerosol-based technique could be used in diagnostic as an MRI contrast agent as well as in anticancer hyperthermia therapy.

### 3.8. Magnetic Heating Efficiency of AeroNPs Dispersion

The most commonly used heating efficiency parameters of magnetic NPs in high-frequency external magnetic fields are the specific loss power (*SLP*) and the intrinsic loss power *(ILP)* [64]. Both the *SLP* and *ILP* parameters are measures of power dissipation in the sample and can be determined by calorimetric measurements, i.e., based on the temperature vs. real-time dependencies. The *SLP* can be expressed as the heating power per unit mass of magnetic material in the sample (usually expressed in W/g), while the *ILP* (measured in Hm${}^{2}$/kg) is used to compare heating efficiency at different experimental conditions including frequency (*f*) and the amplitude of external magnetic field (*H*). The relation between those two parameters goes as follows:

(2) $$ILP=\frac{SLP}{f\cdot H^{2}}$$

To extract the *SLP* and *ILP* from the heating curves the corrected slope method was used [65].

**Figure 14:**
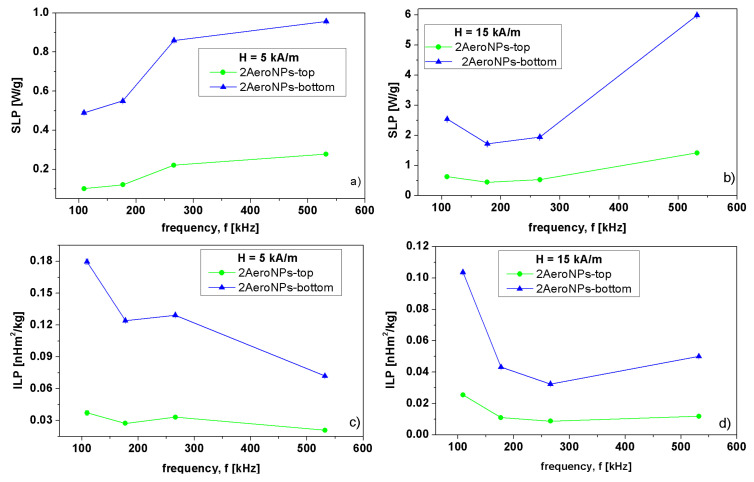
Specific loss power (*SLP*) (**a**,**b**) and intrinsic loss power (*ILP*) (**c**,**d**) as functions of the frequency (*f*) measured in the external magnetic fields *H* = 5 kA/m (left column) and *H* = 15 kA/m (right column) for 2AeroNPs-top (green) and 2AeroNPs-bottom (blue) samples.

It is shown in Figure 14 that *SLP* calculated for different amplitudes of external magnetic field (*H*) increased significantly with the increase of *H* for all used frequencies (*f*). Although the origin of changes in *SLP* for different *H* and *f* is not very clear, it might be related to the variation of the energy dissipation, especially in the case of *H* = 5 kA/m, where the values of *SLP* and *ILP* are relatively small. For *H* = 15 kA/m, the *SLP*(*f*) dependencies were more smooth and the *SLP* showed shallow minima at low frequencies which then increased with the increase of *f*. On the other hand, it was noticeable that *SLP* was larger for the 2AeroNPs-bottom sample than for the 2AeroNPs-top one. This might be due to the presence of larger agglomerates of NPs in the 2AeroNPs-bottom specimen, thus resulting in a larger magnetic moment (*M*) of the sample (as shown in Figure 10). Furthermore, the effect of a slightly higher *d${}_{b}$* than *d${}_{t}$* for dispersions used for measurements should not be neglected. Much lower *SLP* values for studied AeroNPs compared to *SLP* measured for dispersion of bare co-precipitated Fe${}_{3}$O${}_{4}$ nanoparticles [38,39] and these coated with TEA-oleate [9] might be related to the smaller sizes of the 2AeroNPs-top and 2AeroNPs-bottom and their smaller agglomerates formed before coating them with TEA-oleate. The possible factor that might influence the differences in obtained *SLP* is the impact of TEA-oleate coating on the magnetic properties of the nanoparticles. As shown in Figure 10, the TEA-oleate coating caused a significant reduction of the magnetic moment (*M*) of NPs. Furthermore, *SLP* measurements reported in [9,38,39] were performed for NPs dispersed in glycerol, which can alter the relaxation mechanism in NPs subjected to high-frequency external magnetic fields.

The *ILP*(*f*) dependencies were of the same order of magnitude for both *H* = 5 kA/m and *H* = 15 kA/m. In particular, for the 2AeroNPs-bottom sample at *H* = 5 kA/m, the *ILP* was changing from 0.18 to 0.09 nHm${}^{2}$/kg, while at *H* = 15 kA/m, it was the highest for *f* = 109 kHz, reaching ∼0.1 nHm${}^{2}$/kg and then dropping and stabilizing around 0.04 nHm${}^{2}$/kg with an increase of *f*. For the 2AeroNPs-top sample, the *ILP* values were smaller by the order of magnitude than for the 2AeroNPs-bottom and were varying in the range from 0.02 to 0.037 nHm${}^{2}$/kg at *H* = 5 kA/m. Correspondingly, at *H* = 15 kA/m, the *ILP* was changing from ∼0.009 to 0.0255 nHm${}^{2}$/kg.

### 3.9. MR Relaxation Measurements and MR Imaging for the 1AeroNPs and 2AeroNPs Specimens

**Figure 15:**
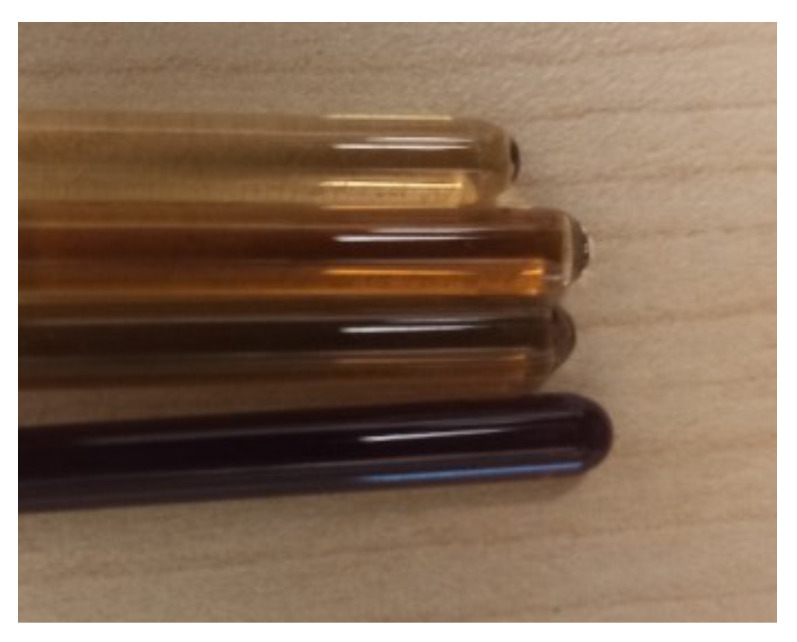
Photograph of phantoms consisting of the NMR tubes containing 1AeroNPs-top dispersions. For the experiment, the concentrations of nanoparticles, 28 mg/mL, 14 mg/mL, 4.67 mg/mL, and 2.8 mg/mL, in the dispersions were used (the darker the color of dispersion in the tube, the higher the concentration of the dispersion).

As mentioned above in Section 2.2, the 1AeroNPs-top and 1AeroNPs-bottom were prepared only for the MRI experiments. For all studied specimens (1AeroNPs-top, 1AeroNPs-bottom, 2AeroNPs-top, and 2AeroNPs-bottom), 140 mg/mL concentrations of dispersions were prepared. Subsequently, they were diluted 5, 10, 30, and 50 times to obtain, respectively, 28 mg/mL, 14 mg/mL, 4.67 mg/mL, and 2.8 mg/mL concentrations of NPs for MR studies (Figure 15).

**Figure 16:**
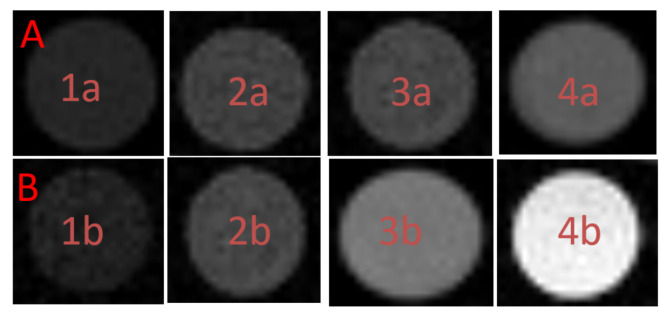
(**A**) An example of a spin echo *T*${}_{2}$-weighted MR image (*TR*/*TE* = 7500 ms/8 ms) of the 1AeroNPs-top (1a, 2a, 3a, 4a) with concentrations of 28 mg/mL (1a), 14 mg/mL (2a), 4.67 mg/mL (3a), and 2.8 mg/mL (4a), respectively (here the darker image of the tube indicates the shorter *T*${}_{2}$, the higher concentration of NPs); (**B**) An example of a *T*${}_{1}$-weighted MR image (*TR*/*TE* = 3 ms/1.5 ms) of the 1AeroNPs-top (1b, 2b, 3b, 4b) with concentrations of 28 mg/mL (1b), 14 mg/mL (2b), 4.67 mg/mL (3b), and 2.8 mg/mL (4b), respectively.

To measure the magnetic response of the NPs, we have used a 9.4T MRI system. The changes in *T*${}_{2}$ and *T*${}_{1}$ relaxation times due to the presence of NPs were demonstrated by collecting the *T*${}_{2}$- and *T*${}_{1}$- weighted MRI images for phantoms with different concentrations of NPs. Figure 16 shows the MR images of a phantom containing various concentrations of 1AeroNPs, obtained with spin echo (*SE*) and *TRUE FISP* pulse sequences, respectively. Different brightening of *T*${}_{2}$- weighted (Figure 16A) and *T*${}_{1}$-weighted (Figure 16B) MR images, with the decreasing concentration of nanoparticles, were observed.

**Figure 17:**
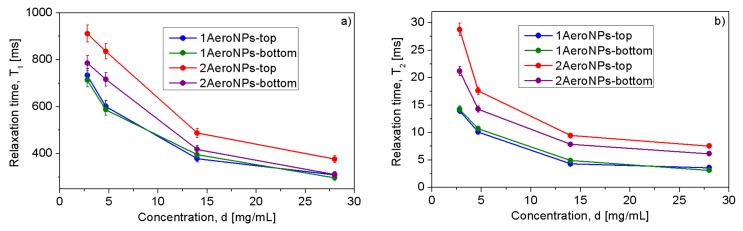
Correlation between *T*${}_{1}$ (**a**) and *T*${}_{2}$ (**b**) relaxation times and concentrations *d* of Fe${}_{3}$O${}_{4}$ NPs in the 1AeroNPs-top and 1AeroNPs-bottom as well as 2AeroNPs-top and 2AeroNPs-bottom samples.

The relationships between relaxation times and concentrations of NPs are shown in Figure 17a,b. For all studied types of NPs, an almost linear increase of *T*${}_{2}$ with the decrease of NP concentrations in their dispersions has been shown.

The differences between the values of *T*${}_{2}$ relaxation times measured for 1AeroNPs-top and 1AeroNPs-bottom dispersions are insignificant. However, they are distinctly shorter than the *T*${}_{2}$ relaxation times measured for 2AeroNPs-top and 2AeroNPs-bottom specimens. The difference in the nanoparticle sizes can explain the differences in relaxation times between 1AeroNPs and 2AeroNPs. In the case of 1AeroNP specimens, the nanoparticles were larger than for the 2AeroNPs. Thus, large NPs more effectively shorten the relaxation times than smaller ones. An interesting phenomenon is a significant difference in the *T*${}_{2}$ relaxation times measured for the 2AeroNPs-top and 2AeroNPs-bottom specimens. The discrepancies may be due to the different distribution of the nanoparticle sizes for “-top” and “-bottom” specimens. As was proven by TEM and XRD studies, the average size of nanoparticles for the 2AeroNPs-top samples was ∼5–6 nm, and they were no larger than 11 nm (Figure 3c). Although the nanoparticles in the 2AeroNPs-bottom also had an average diameter of ∼5–6 nm, the *FWHM* of their distribution reaches 6 nm, which is approximately 2 times larger than that for the 2AeroNPs-top sample. This indicates the presence of a much higher fraction of larger nanoparticles in the 2AeroNPs-bottom dispersion. As the 2AeroNPs sample was separated into two fractions (“-top” and “-bottom”) during centrifugation, in the 2AeroNPs-bottom fraction the larger agglomerates of NPs are present (Figure 4). This might be a reason why their agglomerates may have a stronger effect on the *T*${}_{2}$ relaxation times from the surrounding water protons.

## 4. Conclusions

TEM studies have shown that adjustment of the processing conditions in the aerosol-based method of obtaining magnetite nanoparticles allows the production of relatively uniform NPs with average diameters of ∼5–6 nm only by changing the concentrations of NH${}_{4}$OH and a mixture of FeCl${}_{3}$·6H${}_{2}$O and FeSO${}_{4}$·7H${}_{2}$O salts in their solutions (see Table 1 and Figure 2, Figure 3 and Figure 4). The coating of 2AeroNPs nanoparticles with TEA-oleate and subsequent centrifugation resulted in obtaining two fractions of nanoparticles, which were different in size distributions (Figure 3c and Figure 4c). In particular, the *FWHM* of the particle size distribution decreased to 3.5 nm for the 2AeroNPs-top sample, proving that it is possible to obtain uniform magnetite nanoparticles using the novel aerosol-based technique. The Rietveld refinement of XRD patterns (Table 2) has given quite similar average crystallite diameters (*L*${}_{vol}$) to those obtained from TEM studies, especially for the 2AeroNPs-top sample. The slightly higher (*L*${}_{vol}$) calculated for the 2AeroNPs-bottom sample might be related to its wider crystallite size distributions. Furthermore, DLS hydrodynamic sizes (Figure 7d) and TEM images confirmed changes in the size of agglomerates present in those two dispersions (2AeroNPs-top and 2AeroNPs-bottom). The use of the aerosol-based technique resulted in a relatively large concentration of dispersions (∼0.2 g/mL). This promoted the formation of agglomerates in the initial state of synthesis. Although the citric acid applied in the last stage of producing water dispersion caused neutralization and partial deagglomeration of NPs, one can assume that it was not sufficient for the full detachment of nanoparticles. For this reason, the TEA-oleate coating allowed the stabilization of those small agglomerates rather than separate NPs. Their further centrifugation separated the dispersion into the “-top” and “-bottom” fractions of different agglomerate sizes. As a consequence, these NPs revealed differences in their magnetic properties. Namely, for the 2AeroNPs-top sample the Mössbauer studies have proven its superparamagnetic properties ((Figure 9b). In the case of the 2AeroNPs-bottom sample, the presence of large agglomerates resulted in the rise of exchange interactions between superparamagnetic NPs, which has its reflection in the Mössbauer spectrum (Figure 9a). The microstructure also impacted the reduction of the saturation magnetization of the 2AeroNPs-bottom and 2AeroNPs-top samples (Figure 10).

The presence of agglomerates might be somewhat beneficial for heating those dispersions in high-frequency magnetic fields, due to interactions between NPs causing their total ferrimagnetic behavior. However, due to the low saturation magnetization of these samples, their dispersions subjected to high-frequency external magnetic fields have shown a rather weak *T*(*t*) response. It was found that *SLP* and *ILP* values were relatively low compared to those measured for magnetite NPs co-precipitated using standard procedures [9,38,39,53].

The MTS assay, however, has revealed differences in the viability of the cancer U118 and normal CHO-K1 cells cultured with the nanoparticles coated with TEA-oleate (2AeroNPs-top and 2AeroNPs-bottom). It was found that both types of NPs with a concentration of 20 μg/mL were more toxic for cancer than for normal cells, which is a particularly important result for the assessment of their potential medical applications. Moreover, the toxicity increased with the increase of NP concentration and the culture time. As a result, a higher toxic effect was observed for 2AeroNPs-top than for 2AeroNPs-bottom specimens. This might be related to a higher number of isolated NPs in the 2AeroNPs-top specimens and their smaller agglomerates in their dispersions, thus larger surface areas of NPs being in direct contact with the cells. This was also confirmed by the holotomographic cross-section images (in the Z-axis direction). Moreover, holotomography has shown that in the 2AeroNPs-top specimen, the nanoparticles were evenly distributed throughout the depth of cells. On the other hand, these studies carried out on the 2AeroNPs-bottom specimen revealed the presence of agglomerates on the surfaces of the cells (such agglomeration was not found for the 2AeroNPs-top specimen). It was also shown that the places of interactions were the same in both types of NPs and cells. Furthermore, there are no preferential sites for the accumulation of NPs in cancer and normal cells. Although the accumulation mechanisms of NPs in both normal and cancer cells are similar, their addition to the U118 cancer cells causes the formation of stress fibers (not observed for the CHO-K1 cells). For the CHO-K1 cells, the presence of small and less agglomerated nanoparticles of the 2AeroNPs-top sample initially caused membrane budding (after 3 h of culture). However, a lack of pathological changes was shown after 24 h.

Small agglomerates of the studied NPs have relatively good effects on the MRI signal. Close to a linear relationship between *T*${}_{1}$ relaxation time and concentrations, *d*, of nanoparticles in the 1AeroNPs-top and 1AeroNPs-bottom, as well as in 2AeroNPs-top and 2AeroNPs-bottom samples, were observed. The *T*${}_{1}$ relaxation times measured for dispersions of 1AeroNPs-top and 1AeroNPs-bottom nanoparticles are comparable and shorter than those for 2AeroNPs-top and 1AeroNPs-bottom nanoparticles. The *T*${}_{2}$ vs. *d* dependencies follow the same tendency. For this reason, one can conclude that with the increase in the concentration of nanoparticles, a stronger reduction of *T*${}_{1}$ and *T*${}_{2}$ relaxation times occurs. These results indicate that both types of NPs can be used as the contrast agents in *T*${}_{2}$- and *T*${}_{1}$-weighted MRI studies, where the 1AeroNPs dispersions of larger particle sizes can reduce relaxation time more significantly than smaller nanoparticles of the 2AeroNPs counterparts.

## Figures and Tables

**Table 1:** Processing conditions and amounts of particular reagents used in the aerosol-based synthesis.

Sample Type	FeCl${}_{3}$ · 6H${}_{2}$O (g)	FeSO${}_{4}$ · 7H${}_{2}$O (g)	NH${}_{4}$OH (25%) (g)	Feeding Rate of Salts (mL/s)	Feeding Rate of Ammonia (mL/s)
1AeroNPs	0.459	0.24	11	0.035	0.017
2AeroNPs	0.918	0.48	18	0.035	0.017

**Table 2:** The unit cell parameters (*a*) calculated with the use of Rietveld refinement of the XRD scans for magnetite NPs of all studied specimens; volume-weighted coherently diffracting domain sizes (*L*${}_{vol}$); criteria of fit: *R*${}_{exp}$—*R*-expected, *R*${}_{wp}$—*R*-weighted pattern, and *GOF*—the goodness of fit for Rietveld refinement of XRD patterns.

Sample	*a* (Å)	Space Group	$L_{vol}$ (nm)	$R_{exp}$	$R_{wp}$	*GOF*
Starting values	8.397					
1AeroNPs	8.382	cubic	10.4	0.422	0.497	1.177
2AeroNPs-bottom	8.373	*Fd$\bar{3}$m*	8.4	0.781	0.804	1.030
2AeroNPs-top	8.377		6.8	0.954	0.991	1.039

## Data Availability

All data generated or analyzed during this study are included in this article.
